# Self-Concept Clarity and Loneliness among College Students: The Chain-Mediating Effect of Fear of Negative Evaluation and Self-Disclosure

**DOI:** 10.3390/bs14030194

**Published:** 2024-02-28

**Authors:** Tingting Pang, Haiying Wang, Xinran Zhang, Xianbing Zhang

**Affiliations:** 1 School of Psychology, Northeast Normal University, Changchun 130024, China; pangtt187@nenu.edu.cn (T.P.); wanghy178@nenu.edu.cn (H.W.); psy_zxr@outlook.com (X.Z.); 2 Faculty of Education, Northeast Normal University, Changchun 130024, China

**Keywords:** self-concept clarity, loneliness, fear of negative evaluation, self-disclosure, chain-mediating effect

## Abstract

Self-concept clarity is considered a crucial indicator of mental health. Although several studies have examined the correlation between self-concept clarity and loneliness, few studies have investigated the underlying mechanism. Based on the evolutionary theory of loneliness and social penetration theory, this cross-sectional study focused on cognitive and behavioral factors in interpersonal communication situations, aiming to explore the relationship between self-concept clarity and loneliness as well as its internal mechanism. An online questionnaire survey comprised 1145 college students in China to assess their self-concept clarity, fear of negative evaluation, self-disclosure, and loneliness. After controlling for demographic variables, the results showed that self-concept clarity was not only able to directly and negatively predict loneliness but also indirectly predict loneliness through the independent mediating effects of fear of negative evaluation and self-disclosure, as well as the chain-mediating effect of fear of negative evaluation to self-disclosure. This study provides important implications for cognitive and behavioral intervention strategies to alleviate people’s loneliness and improve their mental health.

## 1. Introduction

Loneliness is a negative psychological experience that arises from genuine interpersonal deficiencies or unfulfilled desires for social connections [1]. According to Erikson’s theory of psychological crisis, the collegiate phase is a time when individuals cultivate interpersonal bonds to attain intimacy while avoiding loneliness. Loneliness is a prevalent issue among Chinese college students, particularly in the context of increased academic pressure and intense social competition [2]. A study conducted to investigate the prevalence of loneliness among college students in a certain province revealed that more than half of the college students experienced feelings of loneliness, with some reporting severe loneliness [3]. Chronic experiences of loneliness can potentially contribute to psychological issues such as anxiety and depression, as well as behavioral disorders such as internet addiction and aggression [4]. Therefore, in order to promote the holistic well-being of college students, it is imperative to investigate the determinants and mechanisms underlying their experience of loneliness.

### 1.1. Self-Concept Clarity and Loneliness

Self-concept clarity, an important determinant of an individual’s psychological adjustment, is closely related to feelings of loneliness. Self-concept clarity refers to the structural aspect of one’s self-concept, encompassing the certainty, and coherence of an individual’s self-perception [5]. Self-doubt, ambivalence, and unclear self-perceptions can give rise to a variety of detrimental psychological problems, including social anxiety, depression, and suicidal tendencies [6,7]. Recent studies have indicated a negative association between self-concept clarity and levels of loneliness [8,9]. Individuals with high self-concept clarity are more likely to understand their own needs, values, and goals, actively seek social support and interactions, which increases relationship stability and quality, and ultimately reduces feelings of loneliness [10]. In addition, longitudinal studies have shown that higher self-concept clarity can predict lower negative interpersonal interactions [11], and the improvement of self-concept is positively correlated with relationship maintenance motivations and behaviors [12]. Conversely, those with low self-concept clarity may experience confusion and uncertainty regarding their identities, roles, and goals. Insufficient self-awareness can cause individuals to feel less confident in social situations and unable to form meaningful connections with others, ultimately increasing the risk of loneliness [13]. Although several studies have explored the relationship between self-concept clarity and loneliness, few studies have investigated the underlying mechanism. As a concept at the level of self-perception, self-concept clarity may also influence feelings of loneliness resulting from interpersonal strains or deficits through other mediating variables.

### 1.2. The Mediating Role of Fear of Negative Evaluation

Fear of negative evaluation is a fundamental aspect of social anxiety. It involves an individual’s apprehension and discomfort regarding potential unfavorable evaluations by others in social contexts [14]. Research has shown a positive association between fear of negative evaluation and loneliness. Individuals who fear negative judgments from their social environment may hesitate to initiate connections, ultimately leading to feelings of isolation over time [15,16]. The evolutionary theory of loneliness suggests that negative self-defeating cognitions contribute to the development of loneliness [17]. Individuals with high levels of fear of negative evaluation believe that others examine their appearance, speech, and behavior against strict standards, which may lead to unfavorable judgments [18]. As a result, fear of negative evaluation, which is a negative perception of social situations, can hinder individuals’ motivation to establish meaningful relationships, thereby promoting experiences of loneliness [19]. 

Besides, fear of negative evaluation is influenced by self-concept clarity. Individuals with lower self-concept clarity tend to be more sensitive to negative evaluations and feedback in social situations [20]. Self-concept clarity also affects how individuals perceive and understand social information. People with low self-concept clarity tend to misinterpret social cues during interpersonal interactions, attributing negative or passive meanings to them [21,22]. Considering the above, it is probable that fear of negative evaluation serves as a mediator between self-concept clarity and loneliness.

### 1.3. The Mediating Role of Self-Disclosure

Self-disclosure is the act of individuals sharing personal information about their thoughts, feelings, and experiences with others [23]. According to the social penetration theory, self-disclosure serves as a vital mechanism for fostering social interactions and is a prerequisite for establishing and maintaining intimate relationships, such as friendships [24]. Research indicates that self-disclosure can help maintain strong interpersonal connections, enhance intimacy among partners, and contribute to greater satisfaction in friendships [25,26]. Strong friendships and interpersonal relationships can play a pivotal role in alleviating feelings of loneliness [27]. Additionally, the information age has allowed individuals to share their thoughts and feelings with friends at any time and place through social networks, thereby receiving positive feedback and social support, which can reduce feelings of isolation [28]. 

Moreover, self-disclosure demonstrates a strong association with self-concept clarity. According to research, an individual’s level of self-disclosure is positively predicted by self-concept clarity, which is stronger than self-esteem when disclosing personal information to their romantic partners [29]. Individuals with low self-concept clarity tend to limit their self-expression due to ambiguous or conflicting self-perceptions, in order to avoid suspicion and misunderstanding from others. Furthermore, there is a significant negative correlation between self-concept clarity and social anxiety. This means that individuals with low self-concept clarity tend to experience higher levels of social anxiety [30]. Those with elevated social anxiety often adopt self-concealment strategies to protect themselves from potential negative evaluations when they perceive deficits in their self-image. This can lead them to engage in fewer conversations and disclose less information to others [31,32]. Consequently, self-disclosure may act as a mediating factor in the relationship between self-concept clarity and loneliness.

### 1.4. The Chain-Mediating Role of Fear of Negative Evaluation and Self-Disclosure

There is a strong correlation between fear of negative evaluation and self-disclosure. According to impression management theory, individuals may employ strategies and modify their behavior to establish or maintain the perception that others hold of them [33]. People tend to measure the success of constructing their self-image by perceiving others’ evaluations and thus decide how much to reveal about themselves [34]. Individuals who experience high levels of fear regarding negative evaluations often believe that others will judge them and provide negative feedback about them. Consequently, in order to cope with interpersonal pressures and mitigate social risks, they limit the disclosure of personal information to conceal their shortcomings and maintain their desired self-image [35]. Although online platforms provide effective means for self-disclosure, their increased visibility and persistent nature increase the potential risk of negative evaluations from others, which subsequently influences the frequency and manner of self-disclosure by individuals on social networks [36,37]. It can be seen that people’s fear of negative evaluation will limit the extent of their self-disclosure. Therefore, self-concept clarity may affect loneliness through the chain-mediating effect of fear of negative evaluation and self-disclosure.

### 1.5. The Current Study

**Figure 1:**
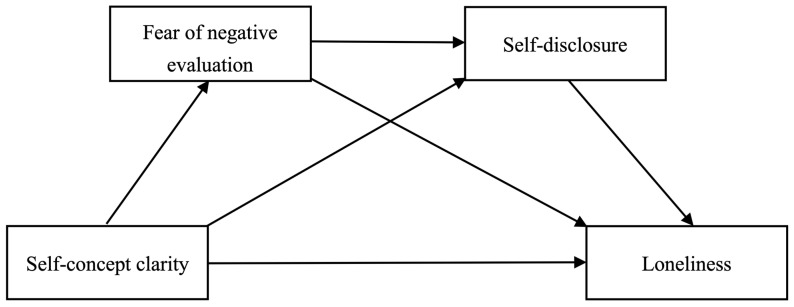
Hypothetical model of the study.

College is a period when individuals form their self-concept and establish their self-identity. Simultaneously, it is also a time when they seek and establish social relationships with others to reduce feelings of loneliness. Although several studies have examined the correlation between self-concept clarity and loneliness [8,9], few have investigated the underlying mechanism. Based on the evolutionary theory of loneliness and social penetration theory, this study adopted a cross-sectional approach and recruited Chinese college students to explore the relationship between self-concept clarity and loneliness, as well as the chain-mediating effect of fear of negative evaluation and self-disclosure. This research aims to reveal the cognitive and behavioral mechanisms of the formation of loneliness in college students, which provides important implications for alleviating people’s loneliness and enhancing their mental health. The study hypothesizes that (Figure 1):

**Hypothesis 1.**  *Self-concept clarity has a negative predictive effect on loneliness.*

**Hypothesis 2.**  *Fear of negative evaluation mediates the relationship between self-concept clarity and loneliness.*

**Hypothesis 3.**  *Self-disclosure also mediates the relationship between self-concept clarity and loneliness.*

**Hypothesis 4.**  *Fear of negative evaluation and self-disclosure play a chain-mediating role between self-concept clarity and loneliness.*

## 2. Materials and Methods

### 2.1. Participants 

This study recruited participants online using the Questionnaire Star platform. The researchers randomly distributed the questionnaire link or QR code to college student subject recruitment groups in Changchun, Jinan, Harbin, and other cities in China. A total of 1300 college students agreed to participate in the study and completed all the questions. After deleting participants with obvious patterns of responses, 1145 (*M* = 21.46 years, *SD* = 1.95, range 18 to 26) valid questionnaires were obtained, with a validity rate of 88%. Among the participants, there were 595 (51.97%) males and 550 (48.03%) females; 911 (79.56%) undergraduates and 234 (20.44%) postgraduates; 609 (53.19%) only child and 536 (46.81%) non-only child; 709 (61.92%) from urban areas and 436 (38.08%) from rural areas.

### 2.2. Measures

#### 2.2.1. Self-Concept Clarity Scale

The Self-Concept Clarity Scale developed by Campbell et al. is used in this study, with the Chinese version of the scale translated by Niu et al. [38]. The scale consists of 12 items (e.g., “I spend a lot of time wondering about what kind of person I really am”), with 10 items reverse-scored, and utilizes a 5-point Likert scale (1—strongly disagree, 2—disagree, 3—neither agree nor disagree, 4—agree, 5—strongly agree). A higher total score indicates a higher level of self-concept clarity. In this study, Cronbach’s alpha coefficient for this scale was found to be 0.88.

#### 2.2.2. Fear of Negative Evaluation Scale

This study employs the “Brief Fear of Negative Evaluation Scale” (BFNES) developed by Leary, with the Chinese version of the scale translated and revised by the domestic researcher Chen Zhiyan [14]. The scale consists of 12 items (e.g., “I am frequently afraid of other people noticing my shortcomings”), with 8 items scored positively and 4 items scored inversely, using a 5-point Likert scale (1—not at all characteristic of me, 2—slightly characteristic of me, 3—moderately characteristic of me, 4—very characteristic of me, 5—extremely characteristic of me). A higher total score indicates a higher level of fear of negative evaluation in individuals. Some researchers suggest that only the positively scored items are effective and recommend using only these 8 items for assessment [39]. Therefore, in this study, only the 8 positively scored items were selected. Cronbach’s alpha coefficient for this scale was found to be 0.95.

#### 2.2.3. Self-Disclosure Scale

The quantitative dimension of the Multidimensional Self-Disclosure Scale was used as a measure of individual self-disclosure [40]. This dimension consists of a total of 7 items (e.g., “I seldom talk about myself to others”), with 4 items scored positively and 3 items scored inversely. A 5-point Likert scale is utilized (1—very inconsistent, 2—somewhat inconsistent, 3—uncertain, 4—somewhat consistent, 5—very consistent), with a higher total score indicating a higher level of self-disclosure by the individual. The scale was translated by psychology faculty members and back-translated by two graduate students to finalize the item content. In this study, the Cronbach’s alpha coefficient for this scale was found to be 0.74.

#### 2.2.4. Loneliness Scale

The measurement of loneliness in this study is based on the UCLA Loneliness Scale, developed by Russell. The Chinese version of the scale was selected from the “Handbook of Psychological Assessment Scales” compiled by Wang Xiangdong and his colleagues [41]. This scale consists of a total of 20 items (e.g., “Do you often feel in harmony with those around you”), with 11 items scored positively and 9 items scored inversely. The scale uses a 4-point Likert scale (1—never, 2—rarely, 3—sometimes, 4—often), with a higher total score indicating a higher level of loneliness in the individual. In this study, the Cronbach’s alpha coefficient for this scale in this study was found to be 0.92.

### 2.3. Procedure and Data Analyses 

This study was conducted using an online questionnaire survey method. The researcher first uploaded the research questionnaire to the Questionnaire Star platform, generated the link and QR code, and then randomly distributed it to college student subject recruitment groups in Changchun, Jinan, Harbin, and other cities in China. The college students completed the online questionnaire by clicking the link or scanning the QR code.

The initial page of the online questionnaire displayed the informed consent form, which informed participants of the survey’s purpose and voluntary and anonymous nature. The lower section provided the option to agree or decline participation in the study. If the participant agreed, they proceeded to the subsequent page to answer the survey’s questions sequentially. If the participant declined, the questionnaire automatically redirected to the end screen. All questionnaires were anonymous, and there were no right or wrong answers. Participants responded based on their actual situations after reading and understanding the instructions. 

The survey period is from September 2022 to November 2022. Prior to the survey, ethical approval was obtained from the Academic Ethics Committee of the College of Psychology of Northeast Normal University. All procedures used in this study were conducted in accordance with general ethical guidelines in psychology.

Descriptive statistics and correlation analysis of variables were processed using SPSS 24.0, and the test of chain-mediation effect was conducted using the Hayes’ macro program PROCESS v3.3 (Model 6) [42].

## 3. Results

### 3.1. Common Method Variance Test

Because the data in this study were collected through self-report measures from participants, the common method variance (CMV) may exist. Harman’s single-factor method analysis was conducted to assess the severity of data homology errors in this study [43]. The results showed that there were six common factors with eigenvalues greater than 1, and the first common factor explaining the variance accounted for 31%, which is lower than the empirical standard point of 40%. Therefore, there was no serious common method variance in the current study.

### 3.2. Descriptive Statistics and Correlation Analysis of Variables

Pearson correlation analysis was conducted for each variable, and the results of the descriptive statistics and correlation analysis are presented in Table 1. The results showed that self-concept clarity exhibited a significant negative correlation with fear of negative evaluation and loneliness, and a significant positive correlation with self-disclosure. Fear of negative evaluation showed a significant positive association with loneliness and a significant negative association with self-disclosure. In addition, self-disclosure was significantly and negatively correlated to loneliness. Because gender showed a significant correlation with fear of negative evaluation, only child status showed a negative correlation with self-disclosure, and place of origin showed correlations with self-concept clarity, self-disclosure, and loneliness, all three variables were used as control variables in the subsequent analysis.

### 3.3. Mediation Effect Analysis

**Figure 2:**
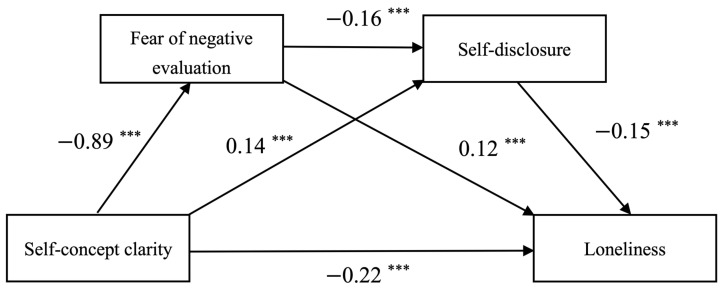
Chain-mediating effect of fear of negative evaluation and self-disclosure (*** *p* < 0.001).

In this study, the SPSS macro program PROCESS v3.3 Model 6 prepared by Hayes was used to conduct a chain-mediation effect test using 5000 bootstrap samples with 95% confidence intervals, controlling for the participants’ gender, only-child status, and place of origin. The results of the regression analysis indicated that self-concept clarity significantly negatively predicted loneliness (*β* = −0.22, *t* = −9.37, *p* < 0.001) and fear of negative evaluation (*β* = −0.89, *t* = −30.48, *p* < 0.001), while it had a significant positive predictive effect on self-disclosure (*β* = 0.14, *t* = 3.98, *p* < 0.001). In addition, fear of negative evaluation exhibited a significant negative predictive effect on self-disclosure (*β* = −0.16, *t* = −6.05, *p* < 0.001) and a positive predictive effect on loneliness (*β* = 0.12, *t* = 6.98, *p* < 0.001). In addition, self-disclosure significantly and negatively predicted loneliness (*β* = −0.15, *t* = −7.47, *p* < 0.001). Detailed results are presented in Table 2 and Figure 2.

As shown in Table 3, further analysis of the mediation effect revealed that fear of negative evaluation and self-disclosure played significant mediating roles in the relationship between self-concept clarity and loneliness. The total mediation effect accounted for approximately 40% of the total effect, which consisted of three pathways. The first pathway was the independent mediation effect of fear of negative evaluation (mediation effect = −0.111, SE = 0.018, bootstrap 95% CI: [−0.145, −0.075]), which accounted for 29.8% of the total effect. The second pathway was the independent mediation effect of self-disclosure (mediation effect = −0.020, SE = 0.007, bootstrap 95% CI: [−0.034, −0.009]), which accounted for 5.5% of the total effect. The third pathway was the chain-mediation effect of fear of negative evaluation and self-disclosure (mediation effect = −0.021, SE = 0.005, bootstrap 95% CI: [−0.032, −0.012]), which accounted for 5.6% of the total effect. Consequently, the results revealed that self-concept clarity not only directly predicted loneliness but also indirectly predicted loneliness through the independent mediating effects of fear of negative evaluation and self-disclosure, as well as through the chain-mediating effect of fear of negative evaluation to self-disclosure, thus supporting our hypothesis.

## 4. Discussion

### 4.1. Effect of Self-Concept Clarity on Loneliness

The study supported Hypothesis 1 by showing that self-concept clarity significantly and negatively predicts college students’ loneliness, which is consistent with previous research [8,9]. Previous empirical studies have also demonstrated a significant negative association between self-concept clarity and depression and social anxiety. Additionally, research has shown that college students with lower self-concept clarity scores are more likely to experience higher levels of depression and social anxiety [30,44]. Self-concept clarity has been found to have a positive correlation with the sense of meaning in life and subjective well-being. Furthermore, it can predict a sense of meaning in life and subjective well-being through the fulfillment of basic psychological needs [45]. These findings emphasize the importance of self-concept clarity as a protective factor in promoting both physical and mental health. Individuals with high self-concept clarity possess a clear and stable self-recognition, which leads to higher levels of self-identity. Conversely, individuals with low self-concept clarity often experience lower self-esteem due to fragmented and incomplete self-knowledge. This can lead to maladaptive behaviors in response to external changes [5]. Therefore, self-concept clarity serves as a negative predictor of loneliness, which is an indicator of social adaptation.

### 4.2. The Mediating Role of Fear of Negative Evaluation and Self-Disclosure

The present study found that fear of negative evaluation served as a mediator in the relationship between self-concept clarity and loneliness, supporting Hypothesis 2. Previous research has indicated that individuals with lower levels of self-concept clarity are more susceptible to higher levels of rejection sensitivity and have greater sensitivity to negative external evaluations [20]. Individuals with low self-concept clarity have difficulty extracting self-relevant information when coping with different situations and rely more on external information for decision-making. This can lead to negative perceptions and attributions in social situations, as they are more likely to perceive external information as stressful and unpredictable. According to the evolutionary theory of loneliness, negative self-defeating perceptions are associated with the development of loneliness [17]. Individuals with a heightened fear of negative evaluation tend to be more sensitive to uncertain events in social settings and perceive them as threatening information [46]. This heightened sensitivity may hinder their motivation to establish meaningful connections with others, ultimately contributing to feelings of loneliness. Consequently, fear of negative evaluation mediates the relationship between self-concept clarity and loneliness.

Furthermore, the study found that self-disclosure plays a mediating role between self-concept clarity and loneliness, which supports Hypothesis 3. Consistent with previous research, the internal consistency of one’s self-concept plays a crucial role in how individuals present themselves to their interpersonal partners, and low self-concept clarity hinders the level of self-disclosure [29]. This finding can be attributed to individuals with low self-concept clarity lacking a clear understanding of themselves, having contradictory and confusing self-information, and experiencing difficulty presenting themselves. Additionally, this finding is consistent with social penetration theory, which suggests that self-disclosure serves as a significant means of maintaining social connections. Individuals with strong friendships and relationships tend to perceive greater social support and receive positive feedback, resulting in increased subjective well-being and reduced feelings of loneliness [47]. Thus, self-disclosure acts as a mediating factor between self-concept clarity and loneliness.

### 4.3. The Chain-Mediating Role of Fear of Negative Evaluation and Self-Disclosure

Finally, this study verifies Hypothesis 4 that self-concept clarity affects loneliness through the chain-mediating effect of fear of negative evaluation and self-disclosure. Fear of negative evaluation can significantly negatively predict fear of self-disclosure. This finding supports the impression management theory that people take actions to establish and maintain a favorable image of themselves in the minds of others [33]. Self-image is often gained through the evaluations of others. Individuals with high fear of negative evaluation typically believe that others will evaluate them negatively, so they reduce or conceal further disclosure of self-relevant information or conceal it in order to maintain their existing self-image [35]. Individuals with low self-concept clarity are more sensitive to information about the external environment because they lack a stable and consistent self-concept [5]. They worry more about possible negative evaluations in social interactions and have a higher fear of negative evaluation. Additionally, individuals with high negative evaluation fear are more likely to adopt avoidance and self-concealment coping styles when dealing with possible stressful social situations [31]. This is not conducive to the disclosure of self-information, and they are unable to express their thoughts in a timely manner. Over time, fear of negative evaluation and reluctance to disclose personal information can hinder the establishment and development of meaningful interpersonal relationships, resulting in increased feelings of loneliness. Therefore, fear of negative evaluation and self-disclosure play a chain-mediating role in the relationship between self-concept clarity and loneliness.

## 5. Implications

The need for belongingness is a fundamental psychological need for individuals. The college phase serves as a crucial period for cultivating interpersonal relationships to satisfy this need and alleviate feelings of loneliness. This cross-sectional survey examined the relationship between self-concept clarity and loneliness among college students. The results suggest that self-concept clarity is a predictor of loneliness, not only through the independent mediating of fear of negative evaluation and self-disclosure but also through a chain-mediating effect of fear of negative evaluation to self-disclosure. This study indicates that individuals with lower self-concept clarity experience more fear and anxiety when facing potential negative evaluations from others in interpersonal situations. They will reduce the intensity of self-disclosure to protect their self-image, which can be detrimental to establishing good interpersonal relationships and may lead to loneliness. The current research enhances our understanding of the underlying mechanisms between self-concept clarity and loneliness and provides new empirical evidence that self-concept clarity is a protective factor for psychological adjustment. Furthermore, it provides theoretical guidance for cognitive and behavioral intervention strategies aimed at promoting the psychological well-being of college students. Therefore, this study suggests that individuals can reduce loneliness by enhancing self-concept clarity through participation in group activities for self-exploration and self-acceptance, conducting cognitive–behavioral training to reduce adverse social cognitions such as fear of negative evaluations, and expanding the level of self-disclosure and increasing social contact through interpersonal training.

## 6. Limitations and Research Perspectives

The results of this study must be considered with several limitations. First, it is important to note that this study may have limited generalizability, because it is based on a specific sample of college students. In order to determine the broader applicability of the findings, further research should include more diverse populations. The second limitation is that the use of a cross-sectional design in this study hinders the establishment of causal relationships. To provide more robust evidence for the proposed mediation model, future studies should consider employing longitudinal or experimental designs that provide a more complete understanding of the dynamic processes and causal relationships. In addition, it is worth considering the potential limitations associated with the use of self-report questionnaires, such as response bias and subjective interpretation. To enhance the reliability and validity of the study, we suggest that future research employ multiple research methods and integrated data collection techniques. For instance, a combination of quantitative and qualitative research methods could be utilized to gain a deeper understanding of the correlation between individuals’ self-concept clarity, fear of negative evaluation, self-disclosure, and loneliness through in-depth interviews or observations. Moreover, further research could investigate additional mediating variables, such as social support and self-esteem, to gain a more comprehensive understanding of the relationship between self-concept clarity and loneliness. Finally, future research could explore the effects of interventions, such as psychoeducation and social skills training, to assist college students in enhancing self-concept clarity, reducing fear of negative evaluation, and increasing self-disclosure, ultimately decreasing loneliness.

## 7. Conclusions

This cross-sectional study finds self-concept clarity not only directly predicts loneliness, but also indirectly through the independent mediating effects of fear of negative evaluation and self-disclosure, as well as the chain-mediating effect of fear of negative evaluation to self-disclosure. This study reveals the mechanism of self-concept clarity on college students’ loneliness from both cognitive (fear of negative evaluation) and behavioral (self-disclosure) perspectives. Accordingly, enhancing self-concept clarity, reducing excessive concerns and anxieties associated with negative evaluations, and increasing the levels of self-disclosure can potentially alleviate loneliness among college students.

## Figures and Tables

**Table 1:** Descriptive statistics and correlation analysis of variables (*n* = 1145).

Variable	1	2	3	4	5	6	7	8
1. Gender	1							
2. Only-Child Status	0.13 **	1						
3. Place of Origin	0.02	0.44 **	1					
4. Age	0.00	−0.12 **	−0.01	1				
5. Self-Concept Clarity	−0.03	−0.01	−0.06 **	0.04	1			
6. Fear of Negative Evaluation	0.12 **	0.02	0.05	0.02	−0.67 **	1		
7. Self-Disclosure	0.04	−0.07 *	−0.08 **	0.01	0.30 **	−0.32 **	1	
8. Loneliness	0.05	0.04	0.06 *	0.04	−0.52 **	0.50 **	−0.36 **	1
*M*	—	—	—	21.46	2.92	3.46	2.87	2.12
*SD*	—	—	—	1.95	0.79	1.05	0.74	0.57
Skewness	—	—	—	0.38	0.57	−0.73	0.40	0.00
Kurtosis	—	—	—	−0.31	−0.33	−0.35	0.03	−0.66
Cronbach’s α	—	—	—	—	0.88	0.95	0.74	0.92

Note. Gender, only child status, place of origin are virtual variables, male = 0, female = 1; only child = 0, non-only child = 1; urban area = 0, rural area = 1; * *p* < 0.05, ** *p* < 0.01.

**Table 2:** Multiple regression analysis (*n* = 1145).

Regression Model		Overall Fitting Index			Regression Coefficient Significance	
Dependent Variable	Independent Variable	*R*	*R* ^2^	*F*	*β*	*t*
Fear of negative evaluation	Gender	0.68	0.46	241.08 ***	0.21	4.63 ***
	Only-child status				−0.01	−0.16
	Place of origin				0.02	0.43
	Self-concept clarity				−0.89	−30.48 ***
Self-disclosure	Gender	0.36	0.13	33.40 ***	0.12	2.76 **
	Only-child status				−0.08	−1.77
	Place of origin				0.06	−1.25
	Self-concept clarity				0.14	3.98 ***
	Fear of negative evaluation				−0.16	−6.05 ***
Loneliness	Gender	0.59	0.34	99.97 ***	0.02	0.78
	Only-child status				0.01	0.33
	Place of origin				0.01	0.45
	Self-concept clarity				−0.22	−9.37 ***
	Fear of negative evaluation				0.12	6.98 ***
	Self-disclosure				−0.15	−7.47 ***

Note. ** *p* < 0.01, *** *p* < 0.001.

**Table 3:** Indirect effects of fear of negative evaluation and self-disclosure.

Path	Effect Value	Boot SE	Boot LLCI	Boot ULCI	Relative Mediation Effect
Path 1: SCC → FNE → LON	−0.111	0.018	−0.145	−0.075	29.8%
Path 2: SCC → SD → LON	−0.020	0.007	−0.034	−0.009	5.5%
Path 3: SCC → FNE → SD → LON	−0.021	0.005	−0.032	−0.012	5.6%
Total indirect effect	−0.152	0.019	−0.190	−0.116	40.9%

Note. SCC = Self-concept clarity, FNE = Fear of negative evaluation, SD = Self-disclosure, LON = Loneliness; Boot SE = Bootstrap standard error, Boot LLCI = Bootstrap lower limit confidence interval, Boot ULCI = Bootstrap upper limit confidence interval.

## Data Availability

The data that support the findings of this study are available on request from the corresponding author.
